# Meta‐Analysis Using Time‐to‐Event Data: A Tutorial

**DOI:** 10.1002/cesm.70041

**Published:** 2025-08-26

**Authors:** Ashma Krishan, Kerry Dwan

**Affiliations:** ^1^ Centre for Biostatistics The University of Manchester, Manchester Academic Health Science Centre Manchester UK; ^2^ Centre for Reviews and Dissemination (CRD) University of York York UK

## Abstract

This tutorial focuses on trials that assess time‐to‐event outcomes. We explain what hazard ratios are, how to interpret them and demonstrate how to include time‐to‐event data in a meta‐analysis. Examples are presented to help with understanding. Accompanying the tutorial is a micro learning module, where we demonstrate a few approaches and give you the chance to practice calculating the hazard ratio. Time‐to‐event micro learning module https://links.cochrane.org/cesm/tutorials/time-to-event-data.

## 1 Introduction

In this tutorial, we explain what time‐to‐event (TTE) data is and what hazard ratios (HR) are, how to interpret them and how they can be included in systematic reviews. A Cochrane review will be used as an example to illustrate time‐to‐event data. The Cochrane review compares daratumumab in addition to antimyeloma medicines to antimyeloma medicines only for adults with newly diagnosed multiple myeloma who are not eligible for a stem cell transplant. The main review question is whether adding the newer medicine, daratumumab to standard antimyeloma treatments helps people live longer or not [1].

## 2 What Is TTE Data?

Survival analysis is used to analyze data where the time until the event is of interest. We call such data TTE data (the time survived until an event, for example, time until tumor recurrence or time until death) [2]. The analysis of TTE data is important when assessing the efficacy of interventions. Oncology represents a major disease area where survival analysis is widely used and drives decision‐making around treatment options. Common practice in the reporting of results from randomized controlled trials (RCTs) is to publish summary statistics for each intervention arm, that is means and standard deviations for continuous outcomes and numerators and denominators for dichotomous outcomes. However, reporting of results for TTE outcomes does not follow these standard statistical methods [3]. TTE data do not usually conform to the normal distribution pattern due to when the events occur. This is because some patients in a given population may experience an event early on, whilst some patients may not experience the event for a longer period of time such as over the course of the trial or beyond the end of the trial. In the Cochrane review, the two TTE outcomes are overall survival (OS), which is the length of time until death from any cause, and progression‐free survival (PFS), which is the length of time until the multiple myeloma progresses (gets worse).

## 3 What Is Censoring?

If the event of interest (death or progression) has not been observed for an individual in a study, then the TTE is censored. Possible reasons for a TTE outcome being censored include data from a study being analyzed at a particular time point, at which the individuals have not experienced the event of interest. Alternatively, the individual could be lost to follow up, that is, the event of interest status at the time of analysis might not be known. In these situations, the only information available on the TTE outcome is the last date on which the individual was known to be event free. In the Cochrane review, for OS everyone included was followed up until either the follow‐up period ended, they did not survive or patients were lost to follow‐up.

## 4 What Is a HR?

The HR is a measure of how many times more or less likely a participant is to experience the event in the intervention group at a particular point in time compared to the control group. In the Cochrane review example, the HR is 0.64 for OS suggesting that patients receiving daratumumab had a 36% (1−0.64 *100%) lower risk of dying compared to patients receiving treatment without daratumumab.

When comparing interventions in a study, an assumption is often made that the HR is constant over time, even though hazards themselves may vary continuously. This is known as the proportional hazards (PH) assumption. The assumption of PH assumes that the hazard rates for the treatment and control groups are constant across the follow‐up period and so the survival curves do not cross. If the PH assumption is violated, please refer to (Section 5.4).

## 5 How Do I Include Time‐to‐Event Data in a Systematic Review?

When TTE data is included in a systematic review with meta‐analysis it is important that the correct effect estimates are extracted from the paper and analyzed. It is important to note that TTE data should not be analyzed using methods for continuous data as these approaches will not take the censoring into account. Dichotomous approaches can also be used as mentioned in the Cochrane handbook, where you need to know the status of all patients at a fixed time point say the proportion of all patients who had an event before 12 months. However, if someone was lost to follow up, it is not known whether they had an event or not so dichotomous approaches can only be used where we know whether everyone has had an event or not.

Below we explain a few approaches that can be used to include the HR and a measure of statistical uncertainty (i.e., variance or standard error [SE]) in a systematic review. All of these approaches are for when individual patient data (IPD) is not available and we are using summary data available from publications. If IPD is available, what to do is explained in (Section 5.5).

### 5.1 Cox PH Model (Or Other Regression Model)

The Cox PH model [4], is the most commonly used method for the analysis of TTE outcomes in RCTs. From the Cox PH model (or other regression models for survival data), we can obtain the HR and the 95% confidence interval (CI). The generic inverse variance approach can be used to perform the meta‐analysis by using the log HR and the SE. Within RevMan [5], the CI or the *p* value can be entered to obtain the SE in the calculator function. The analysis can also be performed in Stata using the metan [6] command, where the reviewers can enter the HR and 95% CI. The analysis can also be performed in R using the meta [7] package to perform a generic inverse variance meta‐analysis.

Trial reports might also report the hazard rates, which can be used to calculate the HR by dividing the hazard rate for the intervention group by the hazard rate for the control group. However, the hazard rates cannot be used to calculate the SE so that would need to be estimated using an alternative method depending on what is reported in the trial report.

### 5.2 Log‐Rank Test

The HR can also be estimated using statistics obtained from performing a log‐rank test. The log‐rank test [8] is another commonly used method for comparing two or more survival curves. The log‐rank approach calculates the following at each event time, the number of events one would expect given the previous event if there were no difference between the groups, for both the intervention and control group. These values are then summed over all event times to give the total expected number of events for the intervention group. The log‐rank test compares observed number of events, to the expected number and can be used to calculate the HR as:

$$\mathrm{HR}=\frac{Observed\;events\;intervention/logrank\;Expected\;events\;intervention}{Observed\;events\;control/logrank\;Expected\;events\;control}$$

$$\text{The variance can be calculated as V}=\frac{1}{[(\frac{1}{Expected\;events\;intervention})+\left(\frac{1}{Expected\;events\;control})\right]}$$

To perform analysis using log rank test we will need the following from a trial report: the observed number of events in both treatment groups and the expected number of events in both treatment groups. An example [9] of this can be seen below: Box 1.

Box 1 **Example of how to calculate the log‐rank test.**

**Table:** 

Observed events in intervention group = 34	Expected events in intervention group = 28
Observed events in control group = 24	Expected events in control group = 29.9
Using these data and equations above we can calculate the HR and V:	
$\mathrm{HR}=\;\frac{34/28}{24/29.9}=1.51V=\;\frac{1}{\left[\left(\frac{1}{28}\right)+\left(\frac{1}{29.9}\right)\right]}=14.46$

Further details are given in Tierney et al. [10] on what to do if the O‐E events are given but the variance is not and how to calculate the variance. It is important that a knowledgeable statistician is consulted if this approach is followed [11].

### 5.3 Digitizing Kaplan−Meier Curves

Another approach for obtaining summary data for TTE analysis is to reconstruct approximate IPD from published Kaplan−Meier (K‐M) curves as suggested by Guyot et al [12]. This approach allows us to obtain pseudo IPD to estimate the HR as well as the opportunity to perform alternative analyses using TTE data.

**Figure 1:**
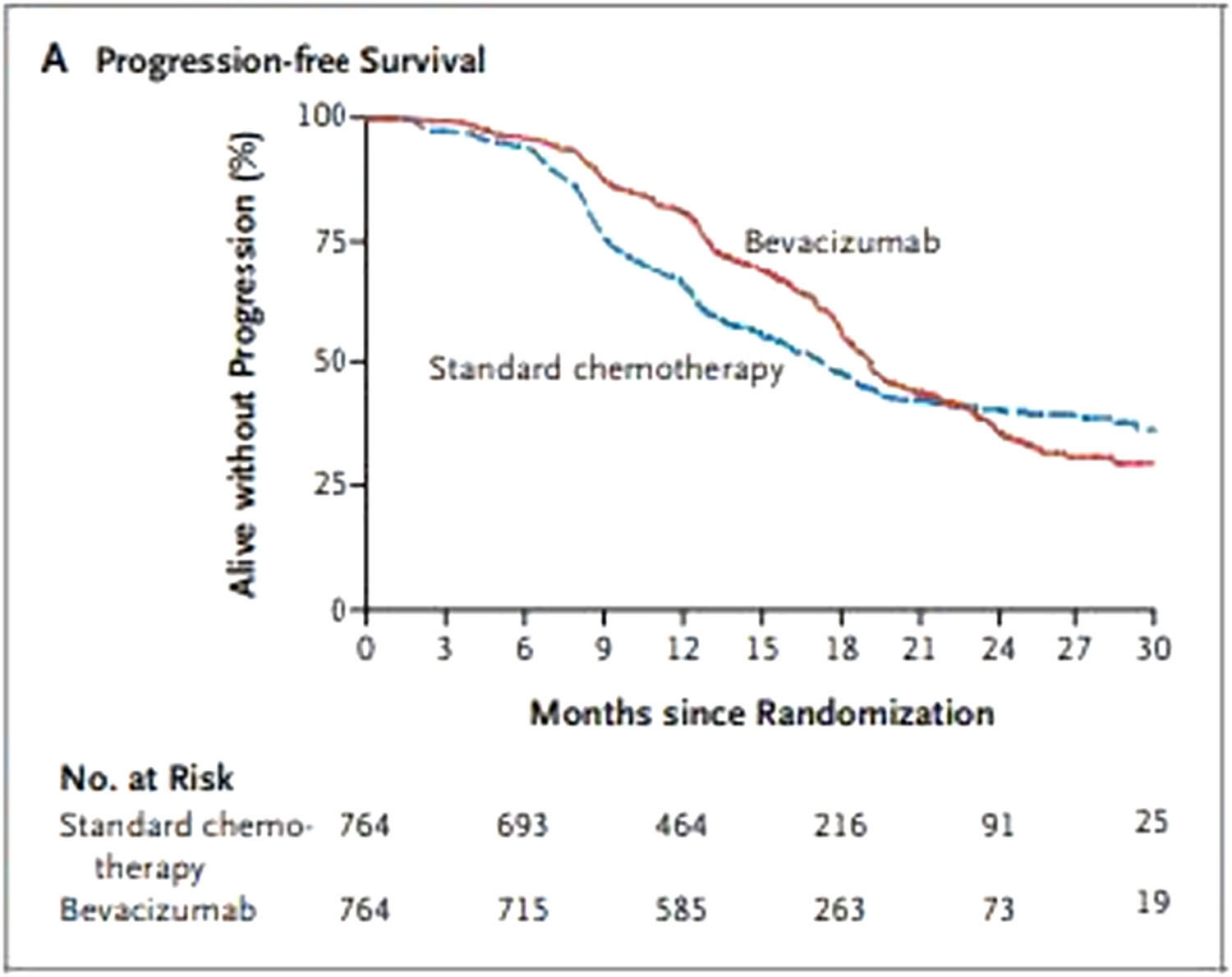
Published Kaplan‐Meier curve.

The following steps are taken for extracting the data from published K‐M curves:

1. Ensure that the survival data is in the correct format as can be seen in the example below.
2. Open software such as DigitizeIt (http://www.digitizeit.de/) or Adobe Illustrator and copy and paste the K‐M plot. We then define what x‐min, x‐max, y‐min and y‐max are. In this example, we can see that x‐min is 0, x‐max is 30, y‐min is 0 and y‐max is 100. To extract data from the graphical images of the published K‐M curves, we click on individual points of the curve using a mouse and the points are recorded as x and y values by the software. If possible, the number of patients at risk for each arm should also be extracted, as the accuracy of the approximated TTE data can be improved by including this information [10].
3. The x and y values should then be saved as CSV files, one for each treatment group curve. The extracted data and the number at risk data which is normally included below the K‐M plot should then be saved in a text file and imported into Stata or R. The text file should contain the data extracted from the x axis and y axis of each curve so time and survival as well as time and number at risk from the risk table.
4. Once the data is extracted, the Stata [13] package, ipdfc [14] or the R [15] package reconstructKM [16] can be used for fitting the Cox model to the reconstructed data. Both of the stats packages, are based on the reconstruction algorithm, which was written by Guyot et al. [12]. A statistician may need to be consulted if this approach is being followed (Figure 1).

### 5.4 What to Do about the PH Assumption?

When fitting a Cox PH model, the PH assumption should always be assessed but previous reviews highlight that the PH assumption is very poorly reported [17, 18]. Although there is no expectation that the review authors should be assessing the PH assumption themselves, if there is any mention or no mention of the PH assumption in the trial paper then this should be stated in the review. This helps with transparency of knowing what has and hasn't been done. If the PH assumption is violated then the results could be over or under estimating the true effect, which would only be apparent once alternative estimation methods have been applied.

### 5.5 What to Do If IPD Is Available?

If IPD is available then two different statistical approaches can be considered, a one‐stage and a two‐stage model. One‐stage models simultaneously analyze IPD from all studies, while accounting for the separate studies. Such models, while more computationally complex, offer additional flexibility to incorporate covariates, interaction terms, or heterogeneity parameters [19, 20]. A series of one‐stage Cox models for the meta‐analysis of TTE data where IPD are available, has been described previously [20].

Alternatively, a two‐stage approach would fit separate models to the data from each study individually as the first stage and then study level results (log HR and SE) from these separate models pooled together using general meta‐analytic models in the second stage. For both approaches it is important to consult a statistician.

#### 5.5.1 Further Reading and Online Content

More information on TTE data, can be found in Chapter 6.8 of The Cochrane Handbook for Systematic Reviews of Interventions [21].

Cochrane Training have produced a micro‐learning module on how to include TTE data in systematic reviews to accompany this article [22].

## Author Contributions

**Ashma Krishan:** conceptualization, writing − original draft, writing − review and editing. **Kerry Dwan:** conceptualization, supervision, writing − review and editing.

## Conflicts of Interest

The authors declare no conflicts of interest.

## 1 Peer Review

The peer review history for this article is available at https://www.webofscience.com/api/gateway/wos/peer-review/10.1002/cesm.70041.

## Data Availability

Data sharing is not applicable to this article as no new data were created or analyzed in this study.

## References

[1] P. Langer , L. John , I. Monsef , et al., “Daratumumab and Antineoplastic Therapy Versus Antineoplastic Therapy Only for Adults With Newly Diagnosed Multiple Myeloma Ineligible for Transplant,” Cochrane Database of Systematic Reviews 5, no. 5 (2024): CD013595, 10.1002/14651858.CD013595.pub2. 38695605 PMC11064765

[2] D. Collett , Modelling Survival Data in Medical Research (A Chapman & Hall Book, 2015). Third Edition.

[3] A. Keech , V. Gebski , and R. Pike , Interpreting and Reporting Clinical Trials. A Guide to the Consort Statement and Principles of Randomised Controlled Trials (Australasian Medical Publishing Company, 2007).

[4] D. R. Cox , “Regression Models and Life‐Tables,” Journal of the Royal Statistical Society Series B: Statistical Methodology 34, no. 2 (1972): 187–202, 10.1111/j.2517-6161.1972.tb00899.x.

[5] Review Manager (RevMan) , Version 8.1.1. The Cochrane Collaboration, 2024.

[6] R. J. Harris , J. J. Deeks , D. G. Altman , M. J. Bradburn , R. M. Harbord , and J. A. C. Sterne , “Metan: Fixed‐ and Random‐Effects Meta‐Analysis,” The Stata Journal: Promoting Communications on Statistics and Stata 8 (2008): 3–28.

[7] S. Balduzzi , G. Rücker , and G. Schwarzer , “How to Perform a Meta‐Analysis With R: A Practical Tutorial,” Evidence Based Mental Health 22 (2019): 153–160. 31563865 10.1136/ebmental-2019-300117 PMC10231495

[8] R. Peto , M. C. Pike , P. Armitage , et al., “Design and Analysis of Randomized Clinical Trials Requiring Prolonged Observation of Each Patient. II. Analysis and Examples,” British Journal of Cancer 35 (1977): 1–39, 10.1038/bjc.1977.1. 831755 PMC2025310

[9] C. Mangioni , G. Bolis , S. Pecorelli , et al., “Randomized Trial in Advanced Ovarian Cancer Comparing Cisplatin and Carboplatin,” JNCI Journal of the National Cancer Institute 81 (1989): 1464–1471, 10.1093/jnci/81.19.1464. 2674459

[10] J. F. Tierney , L. A. Stewart , D. Ghersi , S. Burdett , and M. R. Sydes , “Practical Methods for Incorporating Summary Time‐to‐Event Data Into Meta‐Analysis,” Trials 8 (2007): 16, 10.1186/1745-6215-8-16. 17555582 PMC1920534

[11] T. G. Clark , M. J. Bradburn , S. B. Love , and D. G. Altman , “Survival Analysis Part I: Basic Concepts and First Analyses,” British Journal of Cancer 89, no. 2 (2003): 232–238, 10.1038/sj.bjc.6601118. 12865907 PMC2394262

[12] P. Guyot , A. Ades , M. J. Ouwens , and N. J. Welton , “Enhanced Secondary Analysis of Survival Data: Reconstructing the Data from Published Kaplan−Meier Survival Curves,” BMC Medical Research Methodology 12 (2012): 9, 10.1186/1471-2288-12-9. 22297116 PMC3313891

[13] StataCorp ., Stata Statistical Software: Release 18 (StataCorp LLC, 2023).

[14] Y. Wei and P. Royston , “Reconstructing Time‐to‐Event Data From Published Kaplan−Meier Curves,” Stata Journal: Promoting communications on statistics and Stata 17, no. 4 (2017): 786–802. PMC5796634 29398980

[15] Team RC. R.: A Language and Environment for Statistical Computing. R Foundation for Statistical Computing, Vienna, Austria. Version 3.4.0. 2024, https://www.R-project.org/.

[16] R. Sun . reconstructKM: Reconstruct Individual‐Level Data From Published KM Plots. R package version 0.3.0. 2020, https://CRAN.R-project.org/package=reconstructKM.

[17] S. Batson , G. Greenall , and P. Hudson , “Review of the Reporting of Survival Analyses Within Randomised Controlled Trials and the Implications for Meta‐Analysis,” PLoS One 11, no. 5 (2016): e0154870, 10.1371/journal.pone.0154870. 27149107 PMC4858202

[18] I. Kuitunen , V. T. Ponkilainen , M. M. Uimonen , A. Eskelinen , and A. Reito , “Testing the Proportional Hazards Assumption in Cox Regression and Dealing With Possible Non‐Proportionality in Total Joint Arthroplasty Research: Methodological Perspectives and Review,” BMC Musculoskeletal Disorders 22, no. 1 (May 2021): 489, 10.1186/s12891-021-04379-2. 34049528 PMC8161573

[19] M. C. Simmonds , J. P. T. Higginsa , L. A. Stewartb , J. F. Tierneyb , M. J. Clarke , and S. G. Thompson , “Meta‐Analysis of Individual Patient Data From Randomized Trials: A Review of Methods Used in Practice,” Clinical Trials 2, no. 3 (2005): 209–217, 10.1191/1740774505cn087oa. 16279144

[20] C. T. Smith , P. R. Williamson , and A. G. Marson , “Investigating Heterogeneity in an Individual Patient Data Meta‐Analysis of Time to Event Outcomes,” Statistics in Medicine 24, no. 9 (2005): 1307–1319, 10.1002/sim.2050. 15685717

[21] J. P. T. Higgins , J. Thomas , J. Chandler , et al., *Cochrane Handbook for Systematic Reviews of Interventions* version 6.5 (updated August 2024) (Cochrane, 2024), www.training.cochrane.org/handbook.

[22] Cochrane Training , Including Time‐to‐Event Data in Meta‐Analysis 2025. Content Review Disclaimer.

